# Supplementary material for: Structural basis of the human NAIP/NLRC4 inflammasome assembly and pathogen sensing
Source: Nat Struct Mol Biol. 2024 Jan 4;31(1):82–91. doi: 10.1038/s41594-023-01143-z (PMC10803261; doi:10.1038/s41594-023-01143-z)
Supplement: Supplementary file 1 — Sequence alignment of NAIPs. Residues are highlighted using blue and gray circles with black outlines for the NACHT–NACHT and LRR–LRR interactions with huNLRC4, green circles for the interactions with Needle, and red circles for the interactions with ATP, respectively. Walker A and B motifs are highlighted. [file 41594_2023_1143_MOESM1_ESM.pdf]

nature structural & molecular biology

Article https://doi.org/10.1038/s41594-023-01143-z

Structural basis of the human NAIP/NLRC4 inflammasome assembly and pathogen sensing

In the format provided by the authors and unedited

```
NAIP     MATQQKASDERISQFDHNLLPELSALLGLDAVQLAKELEEEEQKERAKMQKGYNSQMRSE   60
NAIP1    MAEHGESSEDRISEIDYEFLPELSALLGVDAVQLAKSQEEEEHKERMKMKKGFNSQMRSE   60
NAIP2    MAAQGEAVEEIICEFDDDLVSELSTLLRVDALSVLKRQQEEDHKTRMKMKKGFNSQMRSE   60
NAIP5    MAEHGESSEDRISEIDYEFLPELSALLGVDAFQVAKSQEEEEHKERMKMKKGFNSQMRSE   60
NAIP6    MAEHGESSEDRISEIDYEFLAELSARFGMNLVQLAKSQEEEDHKERMKMKKGFNSQMRSE   60
NAIP7    MAEHGESSEDRISEIDYEFLAELSARFGMNLVQLAKSQEEEDHKERMKMKKGFNSQMRSE   60

NAIP     AKRLKTFVTYEPYSSWIPQEMAAAGFYFTGVKSGIQCFCCSLILFGAGLTRLPIEDHKRF   120
NAIP1    AKRLKTFETYDTFRSWTPQEMAAAGFYHTGVKLGVQCFCCSLILFGNSLRKLPIERHKKL   120
NAIP2    AKRLKTFETYDKFRSWTPQEMAAAGFYHTGVKLGVQCFCCSLILFSTRLRKLPIENHKKL   120
NAIP5    AKRLKTFETYDTFRSWTPQEMAAAGFYHTGVRLGVQCFCCSLILFGNSLRKLPIERHKKL   120
NAIP6    AKRLKTFESYDTFRSWTPQEMAAAGFYHTGVKLGVQCFCCSLILFGNSLRKLPIERHKKL   120
NAIP7    AKRLKTFESYDTFRSWTPQEMAAAGFYHTGVKLGVQCFCCSLILFGNSLRKLPIERHKKL   120

NAIP     HPDCGFLLNKDVGNIAKYDIRVKNLKSRLRGGKMRYQEEEARLASFRNWPFYVQGISPCV   180
NAIP1    RPECEFLQGKDVGNIGKYDIRVKSPEKMLRGGKARYHEEEARLESFEDWPFYAHGTSPRV   180
NAIP2    RPECEFLLGKDVGNIGKYDIRVKSPEKMLRGDKARYHEEEARLESFEDWPFYAHGTSPRV   180
NAIP5    RPECEFLQGKDVGNIGKYDIRVKRPEKMLRGGKARYHEEEARLESFEDWPFYAHGTSPRV   180
NAIP6    RPECEFLQGKDVGNIGKYDIRVKSPEKMLRGGKARYHEEEARLESFEDWPFYAHGTSPRA   180
NAIP7    RPECEFLQGKDVGNIGKYDIRVKSPEKMLRGGKARYHEEEARLESFEDWPFYAHGTSPRV   180

NAIP     LSEAGFVFTGKQDTVQCFSCGGCLGNWEEGDDPWKEHAKWFPKCEFLRSKKSSEEITQYI   240
NAIP1    LSAAGFVFTGKRDTVQCFSCGGSLGNWEEGDDPWKEHAKWFPKCEFLQSKKSSEEIAQYI   240
NAIP2    LSAAGFVFTGKRDTVQCFSCGGCLGNWEEGDDPWKEHAKWFPKCEFLQSKKSPEEITQYV   240
NAIP5    LSAAGFVFTGKRDTVQCFSCGGSLGNWEEGDDPWKEHAKWFPKCEFLQSKKSSEEIAQYI   240
NAIP6    LSAAGFVFTGKRDTVQCFSCGGSLGNWEEGDDPWKEHAKWFPKCEFLQSKKSSEEIAQYI   240
NAIP7    LSAAGFVFTGKRDTVQCFSCGGSLGNWEEGDDPWKEHAKWFPKCEFLQSKKSSEEIAQYI   240

NAIP     QSYKGFVDITGEHFVNSWVQRELPMASAYCNDSIFAYEELRLDSFKDWPRESAVGVAALA   300
NAIP1    QGYEGFVHVTGEHFVNSWVRRELPMVSAYCNDSVFANEELRMDTFKDWPHESPVAVDALV   300
NAIP2    QSYEGFLHVTGEHFVNSWVRRELPMVSAYCNDSVFANEELRMDTFKDWPHESPGAVEALV   300
NAIP5    QSYEGFVHVTGEHFVKSWVRRELPMVSAYCNDSVFANEELRMDMFKDWPQESPVGVEALV   300
NAIP6    QDYEGFVHVTGEHFVKSWVRRELPMVSAYCNDSVFTNEELRMDMFKDWPQESPVGFEALV   300
NAIP7    QGYEGFVHVTGEHFVKSWVRRELPMVSAYCNDSVFANEELRMDMFKDWPQESPVGVEALV   300

NAIP     KAGLFYTGIKDIVQCFSCGGCLEKWQEGDDPLDDHTRCFPNCPFLQNMKSSAEVTPDLQS   360
NAIP1    RAGLFYTGKKGIVQCFSCGGCMEKCTEGDDPIQEHNKFFPNCVFLQTPKSSAEVIPALQS   360
NAIP2    KAGLFYTGKRDIVQCFSCGGCMEKWAEGDNPIEDHTKFFPNCVFLQTLKSSAEVIPALQS   360
NAIP5    RAGFFYTGKKDIVRCFSCGGCLEKWAEGDDPMEDHIKFFPECVFLQTLKSSAEVIPTLQS   360
NAIP6    RAGFFYTGKKDIVRCFSCGGCLEKWAEGDDPMEDHIKFFPECVFLQTLKSSAEVIPTLQS   360
NAIP7    RAGFFYTGKKDIVRCFSCGGCLEKWAEGDDPMEDHIKFFPECVFLQTLKSSAEVIPTLQS   360

NAIP     RGELCELLETTSESNLEDSIAVGPIVPE--------------------------------   388
NAIP1    HCALPEAMETTSESNHDDPAAVHSTVVG--------------------------------   388
NAIP2    HCALPEAMETTSESNHDDAAAVHSTVVDVSPSEAQELEPASSLVSVLCRDQDHSEAQGRG   420
NAIP5    QYALPEATETTRESNHGDAAAVHSTVVD--------------------------------   388
NAIP6    QYALPEATETTRESNHDDAAAVHSTVVD--------------------------------   388
NAIP7    QYALPEATETTRESNHGDAAAVHSTVVD--------------------------------   388

NAIP     ------------MAQGEAQWFQEAKNLNEQLRAAYTSASFRHMSLLDISSDLATDHLLGC   436
NAIP1    ------------LGRSEAQWFQEARSLSEQLRDNYTKATFRHMNLPEVCSSLGTDHLIGC   436
NAIP2    CASSGTYLPSTDLGQSEAQWLQEARSLSEQLRDTYTKATFRHMNLPEVYSSLGTDHLLSC   480
NAIP5    ------------LGRSEAQWFQEARSLSEQLRDNYTKATFRHMNLPEVCSSLGTDHLLSC   436
NAIP6    ------------LGRSEAQWFQEARSLSEQLRDTYTKTSFCHMNLPEVCSSLGTDHLLGC   436
NAIP7    ------------LGRSEAQWFQEARSLSEQLRDTYTKTSFCHMNLPEVCSSLGTDHLLSC   436
```

```
                                            Walker A
NAIP    DLSIASKHISKPVQEPLVLPEVFGNLNSVMCVEGEAGSGKTVLLKKIAFLWASGCCPLLN    496
NAIP1   DVSIISKHISQPVQGALTIPEVFSNLSSVMCVEGETGSGKTTFLKRIAFLWASGCCPLLY    496
NAIP2   DVSIISKHISQPVQGSLTIPEVFSNLNSVMCVEGEAGSGKTTFLKRIAFLWASGCCPLLY    540
NAIP5   DVSIISKHISQPVQEALTIPEVFSNLNSVMCVEGETGSGKTTFLKRIAFLWASGCCPLLY    496
NAIP6   DVSIISKHVSQPVQGALTIPEVFSNLSSVMCVEGEAGSGKTTFLKRIAFLWASGCCPLLY    496
NAIP7   DVSIISKHISQPVQGALTIPEVFSNLSSVMCVEGEAGSGKTTFLKRIAFLWASGCCPLLY    496

                                                         Walker B
NAIP    RFQLVFYLSLSSTRPDEGLASIICDQLLEKEGSVTEMCVRNIIQQLKNQVLFLLDDYKEI    556
NAIP1   RFQLVFYLSLSSITPDQGLANIICAQLLGAGGCISEVCLSSIIQQLQHQVLFLLDDYSGL    556
NAIP2   RFQLVFYLSLSSITPGQELAKIICAQLLGAGGCISEVCLSSIIQQLQHQVLFLLDDYSGL    600
NAIP5   RFQLVFYLSLSSITPDQGLANIICAQLLGAGGCISEVCLSSSIQQLQHQVLFLLDDYSGL    556
NAIP6   RFQLVFYLSLSSITPDQGLANIICTQLLGAGGCISEVCLSSSIQQLQHQVLFLLDDYSGL    556
NAIP7   RFQLVFYLSLSSITPDQGLDNIICTQLLGAGGCISEVCLSSSIQQLQHQVLFLLDDYSGL    556

NAIP    CSIPQVIGKLIQKNHLSRTCLLIAVRTNRARDIRRYLETILEIKAFPFYNTVCILRKLFS    616
NAIP1   ASLPQALHTLITKNYLSRTCLLIAVHTNRVRGIRSYLDTSLEIKEFPLSNTVYILKKFFS    616
NAIP2   ASLPQALHTLITKNYLSRTCLLIAVHTNKVRGIRPYLDTSLEIKEFPFYNTVSVLRKLFS    660
NAIP5   ASLPQALHTLITKNYLSRTCLLIAVHTNRVRDIRLYLGTSLEIQEFPFYNTVSVLRKFFS    616
NAIP6   ASLPQALHTLITKNYLFRTCLLIAVHTNRVRDIRPYLGTSLEIQEFPFYNTVFVLRKFFS    616
NAIP7   ASLPQALHTLITKNYLFRTCLLIAVHTNRVRDIRPYLGTSLEIQEFPFYNTVFVLRKFFS    616

NAIP    HNMTRLRKFMVYFGKNQSLQKIQKTPLFVAAICAHWFQYPFD-PSFDDVAVFKSYMERLS    675
NAIP1   HNIKRLLEFMVYFGQNEDLQGIHKTPLFVAAVCTDWFENPSD-QPFQDMALFKSYMQYLS    675
NAIP2   HDIMRVRKFINYFGHEELQGIHKTPLFVAAVCTDWFKNPSD-QPFQDVALFKAYMQYLS     719
NAIP5   HDIICVEKLIIYFIDNKDLQGVYKTPLFVAAVCTDWIQNASAQDKFQDVTLFQSYMQYLS    676
NAIP6   HDIICVEKLIIYFSENKDLQGVYKTPLFVAAVCNDWNQNASAQDDFQDVTLFHSYMQYLS    676
NAIP7   HDIICVEKLIIYFSENKDLQGVYKTPLFVAAVCNDWNQNASAQDDFQDVTLFHSYMQYLS    676

NAIP    LRNKATAEILKATVSSCGELALKGFFSCCFEFNDDDLAEAGVDEDEDLTMCLMSKFTAQR    735
NAIP1   LKHKGAAKPLQATVSSCGQLALTGLFSSCFEFNSDDLAEAGVDEDEELTTCLMSKFTAQR    735
NAIP2   LKHKGAAKPLQATVSSCGQLALTGLFSSCFEFNSDNLAEAGVDEDEELTTCLMSKFTAQR    779
NAIP5   LKYKATAEPLQATVSSCGQLALTGLFSSCFEFNSDDLAEAGVDEDEKLTTLLMSKFTAQR    736
NAIP6   LKYKATAESLQATVSSCGQLALTGLFSSCFEFNSDDLAEAGVDEDVKLTTFLMSKFTAQR    736
NAIP7   LKYKATAESLQATVSSCGQLALTGLFSSCFEFNSDDLAEAGVDEDVKLTTFLMSKFTAQR    736

NAIP    LRPFYRFLSPAFQEFLAGMRLIELLDSDRQEHQDLGLYHLKQINSPMMTVSAYNNFLNYV    795
NAIP1   LRPVYRFLGPLFQEFLAAMRLTELLSSDRQEDQDLGLYYLRQINSPLKALTTYNNFLKYV    795
NAIP2   LRPVYRFLGPLFQEFLAAVRLTELLSSDRQEDQDLGLYYLRQINSPLKAMSIYHTFLKYV    839
NAIP5   LRPVYRFLGPLFQEFLAAVRLTELLSSDRQEDQDLGLYYLRQIDSPLKAINSFNIFLYYV    796
NAIP6   LRPVYRFLGPLFQEFLAAVRLTELLSSDRQEDQDLGLYYLRQIDSPLKAINSFNIFLYYV    796
NAIP7   LRPVYRFLGPLFQEFLAAVRLTELLSSDRQEDQDLGLYYLRQIDSPLKAINSFNIFLYYV    796

NAIP    SSLPSTKAGPKIVSHLLHLVDNKESLENISENDDYLKHQPEISLQMQLLRGLWQICPQAY    855
NAIP1   FSHPSSKAGPTVVSHLLHLVDETELLENTYKNEDYVNHPPGTSRIMKGLKELWLLSPEYY    855
NAIP2   SSHPSSKAAPTVVSHLLQLVDEKESLENMSENEDYMKLHPEALLWIECLRGLWQLSPESF    899
NAIP5   SSHSSSKAAPTVVSHLLQLVDEKESLENMSENEDYMKLHPQTFLWFQFVRGLWLVSPESS    856
NAIP6   SSHSSSKAAPTVVSHLLQLVDEKESLENMSENEDYMKLHPQTFLWFQFVRGLWLVSPESF    856
NAIP7   SSHSSSKAAPTVVSHLLQLVDEKESLENMSENEDYMKLHPQTFLWFQFVRGLWLVSPESF    856

NAIP    FSMVSEHLLVLALKTAYQSNTVAACSPFVLQFLQGRTLTLGALNLQYFFDHPESLSLLRS    915
NAIP1   SSFVSEHLLRIALNFAYESNTVAECSPFILQFLRGRTLALKVLNLQYFRDHPESLLLVKS    915
NAIP2   SLFISENLLRICLNFAHESNTVAACSPVILQFLRGRTLDLKVLSLQYFWDHPETLLLLKS    959
NAIP5   SSFVSEHLLRLALIFAYESNTVAECSPFILQFLRGKTLALRVLNLQYFRDHPESLLLLRS    916
NAIP6   SSFVSEHLLRLALIFAYESNTVAECSPFILQFLRGRTLALRVLNLEYFWDHPESLLLLRS    916
NAIP7   SSFVSEHLLRLALIFAYESNTVAECSPFILQFLRGRTLALRVLNLEYFWDHPESLLLLRS    916
```

```
NAIP     IHFPIRGNKTSPRAHFSVLETCFDKSQVPTIDQDYASAFEPMNEWERNLAEKEDNVKSYM    975
NAIP1    LEVSINGNKVPKVVDYSVMEKSFETLQPPTIDQDYASAFEQMKEHEKNLSENEETIKSIK    975
NAIP2    IKISLNGNNWVQRIDFSLIEKSFEKVQPPTIDQDYAIAFQPINEVQKNLSEKKHIIKKYE    1019
NAIP5    LKVSINGNKMSSYVDYSF-KTYFENLQPPAIDEEYTSAFEHISEWRRNFAQDEEIIKNYE    975
NAIP6    LKVSINGNKMSSYVDYSF-KTYFENLQPPAINEEYTSAFEHVSEWRRNFAQDEEIIKNYE    975
NAIP7    LKVSINGNKMSSYVDYSF-KTYFENLQPPAINEEYTSAFEHVSEWRRNFAQDEEIIKNYE    975

NAIP     DMQRRASPDLSTGYWKLSPKQYKIPCLEVDVNDIDVVGQDMLEILMTVFSASQRIELHLN    1035
NAIP1    NIFPLQPPKISSGYWKLSPKPCKIPRLEVGVTNMGPADQALLQVLMEVFSASQSIEFRLS    1035
NAIP2    DMKHQIPLNISTGYWKLSPKPYKIPKLEVQVTNTGPADQALLQVLMEVFSASQSIEFRLS    1079
NAIP5    NIRPRALPDISEGYWKLSPKPCKIPKLEVQVNNTDAADQALLQVLMEVFSASQSIEFRLF    1035
NAIP6    NIWPRALPDISEGYWNLSPKPCKIPKLEVQVNNMGPADQALLQVLMEVFSASQSIEFHLF    1035
NAIP7    NIWPRALPDISEGYWNLSPKPCKIPKLEVQVNNMGPADQALLQVLMEVFSASQSIEFHLF    1035

NAIP     HSRGFIESIRPALELSKASVTKCSISKLELSAAEQELLLTLPSLESLEVSGTIQSQDQIF    1095
NAIP1    DSSGFLESIRPALELSKASVTKCSMSRLELSRAEQELLLTLPALQSLEVSETNQLPDQLF    1095
NAIP2    DSSGFLESIRPALELSKASVTKCSMSRLELSREDQKLLLTLPTLQSLEVSETNQLPDQLF    1139
NAIP5    NSSGFLESICPALELSKASVTKCSMSRLELSRAEQELLLTLPALQSLEVSETNQLPEQLF    1095
NAIP6    NSSGFLESIRPALELSKASVTKCSMSRLELSRAEQELLLTLPALQSLEVSETNQLPDQLF    1095
NAIP7    NSSGFLESIRPALELSKASVTKCSMSRLELSRAEQELLLTLPALQSLEVSETNQLPDQLF    1095

NAIP     PNLDKFLCLKELSVDLEGNINVFSVIPEEFPNFHHMEKLLIQISAEYDPSKLVKLIQNSP    1155
NAIP1    HNLHKFLGLKELCVRLDGKPDVLSVLPGEFPNLLHMEKLSIRTSMESDLSKLVKLIQNSP    1155
NAIP2    HNLHKFLGLKELCVRLDSKPDVLSVLPGEFPNLHHMEKLSIRTSTESDLSKLVKLIQNSP    1199
NAIP5    HNLHKFLGLKELCVRLDGKPNVLSVLPREFPNLLHMEKLSIQTSTESDLSKLVKFIQNFP    1155
NAIP6    HNLHKFLGLKELCVRLDGKPDVLSVLPEEFLNLHHMEKLSIRTSTESDLSKLVKFIQNFP    1155
NAIP7    HNLHKFLGLKELCVRLDGKPDVLSVLPEEFLNLHHMEKLSIRTSTESDLSKLVKFIQNFP    1155

NAIP     NLHVFHLKCNFFSDFGSLMTMLVSCKKLTEIKFSDSFFQAVPFVASLPNFISLKILNLEG    1215
NAIP1    NLHVFHLKCDFLSNCDSLMAVLASCKKLREIEFSGRCFEAMPFVNILPNFISLKILNLIS    1215
NAIP2    NLHVFHLKCNFLSNCEPLMTVLASCKKLREIEFSGRCFEAMTFVNILPNFVFLKILNLRD    1259
NAIP5    NLHVFHLKCDFLSNCESLMAVLASCKKLREIEFSGRCFEAMTFVNILPNFVSLKILNLKD    1215
NAIP6    NLHVFHLKCDFLSNCESLMTALASCKKLREIEFSGQCFEAMTFVNILPNFVSLKILSLKG    1215
NAIP7    NLHVFHLKCDFLSNCESLMTALASCKKLREIEFSGQCFEAMTFVNILPNFVSLKILSLKG    1215

NAIP     QQFPDEETSEKFAYILGSLSNLEELILPTGDGIYRVAKLIIQQCQQLHCLRVLSFFKTLN    1275
NAIP1    QQFPDKETSEKFAQALGSLRNLEELLVPTGDGIHQVAKLIVRQCLQLPCLRVLAFHYILD    1275
NAIP2    QQFPDKETSEKFAQALGSLRNLEKLFVPTGDGIHQVAKLIVRQCLQLPCLRVLVFAETLD    1319
NAIP5    QQFPDKETSEKFAQALGSLRNLEELLVPTGDGIHQVAKLIVRQCLQLPCLRVLTFHDILD    1275
NAIP6    QQFADKETSEKFAQALGSLRNLEELLVPTGDGIHQVAKLIVRQCLQLPCLRVLAFHDILD    1275
NAIP7    QQFADKETSEKFAQALGSLRNLEELLVPTGDGIHQVAKLIVRQCLQLPCLRVLAFHDILD    1275

NAIP     DDSVVEIAKVAISGGFQKLENLKLSINHKITEEGYRNFFQALDNMPNLQELDISRHFTEC    1335
NAIP1    NDSVIEIARVATSGGFQKLEKLDLSMNHKITEEGYRNFFQALDNLPNLQNLNICRHIPEC    1335
NAIP2    DDSVLEIAKGATRGGFQKLENLDLTLNHKITEEGYRNFFQVLDNLPNLKNLDISRHIPEC    1379
NAIP5    DDSVIEIARAATSGGFQKLENLDISMNHKITEEGYRNFFQALDNLPNLQELNICRNIPGR    1335
NAIP6    DESVIEIARAATSGSFQKLENLDISMNHKITEEGYRNFFQALDNLPNLQMLNICRNIPGR    1335
NAIP7    DESVIEIG-AATSGSFQKLENLDISMNHKITEEGYRNFFQALDNLPNLQMLNICRNIPGR    1334

NAIP     IKAQATTVKSLSQCVLRLPRLIRLNMLSWLLDADDIALLNVMKERHPQSKYLTILQKWIL    1395
NAIP1    IQVQATTVKALGQCVSRLPSLTRLHMLSWLLDEEDMKVINDVKERHPQSKRLIIFWKWIV    1395
NAIP2    IQIQAITVKALGQCVSRLPSLTRLGMLSWLLDEEDIKVINDVKERHPQSKRLTVHWRWVV    1439
NAIP5    IQVQATTVKALGQCVSRLPSLIRLHMLSWLLDEEDMKVINDVKERHPQSKRLIIFWKLIV    1395
NAIP6    IQVQATTVKALGHCVSRLPSLTRLGMLSWLLDEEDMKVINDVKERHPQSKRLTIFWKWIV    1395
NAIP7    IQVQATTVKALCHCVSRLPSLTRLGMLSWLLDEEDMKVINDVKERHPQSKRLTIFWKWIV    1394
```

NAIP	PFSPIIQK	1403
NAIP1	PFSPVVLE	1403
NAIP2	PFSPVIQK	1447
NAIP5	PFSPVILE	1403
NAIP6	PFSPVVLE	1403
NAIP7	PFSPVVLE	1402
