# Supplementary material for: Structural basis of the human NAIP/NLRC4 inflammasome assembly and pathogen sensing
Source: Nat Struct Mol Biol. 2024 Jan 4;31(1):82–91. doi: 10.1038/s41594-023-01143-z (PMC10803261; doi:10.1038/s41594-023-01143-z)
Supplement: Supplementary file 2 — Reporting Summary [file 41594_2023_1143_MOESM2_ESM.pdf]

nature portfolio

Corresponding author(s): Xiaodi Yu

Last updated by author(s): Aug 22, 2023

Reporting Summary

Nature Portfolio wishes to improve the reproducibility of the work that we publish. This form provides structure for consistency and transparency in reporting. For further information on Nature Portfolio policies, see our Editorial Policies and the Editorial Policy Checklist.

Statistics

For all statistical analyses, confirm that the following items are present in the figure legend, table legend, main text, or Methods section.

n/a	Confirmed	
☐	☒	The exact sample size (*n*) for each experimental group/condition, given as a discrete number and unit of measurement
☐	☒	A statement on whether measurements were taken from distinct samples or whether the same sample was measured repeatedly
☒	☐	The statistical test(s) used AND whether they are one- or two-sided<br>*Only common tests should be described solely by name; describe more complex techniques in the Methods section.*
☒	☐	A description of all covariates tested
☒	☐	A description of any assumptions or corrections, such as tests of normality and adjustment for multiple comparisons
☒	☐	A full description of the statistical parameters including central tendency (e.g. means) or other basic estimates (e.g. regression coefficient) AND variation (e.g. standard deviation) or associated estimates of uncertainty (e.g. confidence intervals)
☒	☐	For null hypothesis testing, the test statistic (e.g. *F*, *t*, *r*) with confidence intervals, effect sizes, degrees of freedom and *P* value noted<br>*Give P values as exact values whenever suitable.*
☒	☐	For Bayesian analysis, information on the choice of priors and Markov chain Monte Carlo settings
☒	☐	For hierarchical and complex designs, identification of the appropriate level for tests and full reporting of outcomes
☒	☐	Estimates of effect sizes (e.g. Cohen's *d*, Pearson's *r*), indicating how they were calculated

Our web collection on statistics for biologists contains articles on many of the points above.

Software and code

Policy information about availability of computer code

Data collection	EPU 3.3.0
Data analysis	CryoSparc 4.0, Chimera 1.18, Phenix 1.18.2-3874, Coot 0.9.6, Pymol 2.5.0, Rosetta 3.13, Schrodinger 2021-1, MolProbity, EMRinger, Swiss-model 2019

For manuscripts utilizing custom algorithms or software that are central to the research but not yet described in published literature, software must be made available to editors and reviewers. We strongly encourage code deposition in a community repository (e.g. GitHub). See the Nature Portfolio guidelines for submitting code & software for further information.

Data

Policy information about availability of data

All manuscripts must include a data availability statement. This statement should provide the following information, where applicable:

- Accession codes, unique identifiers, or web links for publicly available datasets
- A description of any restrictions on data availability
- For clinical datasets or third party data, please ensure that the statement adheres to our policy

The cryo-EM maps of the human NLRC4 C11, C12 and Needle/huNAIP/huNLRC4R288A have been deposited with accession codes (PDB-8FW2, EMD-29496), (PDB-8FW9, EMD-29498) and (PDB-8FVU, EMD-29493), respectively. Previously published models, used to interpret human NLRC4 and NAIP structures, were

presented in this study along with their corresponding PDB ID codes and references. Other data are available from the corresponding author upon reasonable request.

Human research participants

Policy information about studies involving human research participants and Sex and Gender in Research.

Reporting on sex and gender	n/a
Population characteristics	n/a
Recruitment	n/a
Ethics oversight	n/a

Note that full information on the approval of the study protocol must also be provided in the manuscript.

Field-specific reporting

Please select the one below that is the best fit for your research. If you are not sure, read the appropriate sections before making your selection.

☒ Life sciences ☐ Behavioural & social sciences ☐ Ecological, evolutionary & environmental sciences

For a reference copy of the document with all sections, see nature.com/documents/nr-reporting-summary-flat.pdf

Life sciences study design

All studies must disclose on these points even when the disclosure is negative.

Sample size	A significant number of cryo-EM datasets (n=6) were collected to achieve the necessary resolutions for modeling. In our case, resolution limit was attained for the CARD domain because of flexibility issues. For all biochemical experiments, sample size was determined as described in the Methods section, whenever applicable.
Data exclusions	Particles were automatically picked. Images with contaminations were deselected. During the process of classification and refinement, denatured or damaged protein particles were removed. For the biochemical experiments, no data were excluded from our analyses.
Replication	Inflammasome assembly, purification, and analysis of cryo-EM samples were repeated at least three times.
Randomization	This study is not involved in Animal or clinical trials. No randomization was performed. All particles in the raw images were selected and analyzed to ensure a unbiased initial particle population. After removal of damaged particles, the dataset is randomly separated into two subsets that are analyzed separately to determine the spatial resolution.
Blinding	This study is not involved in Animal or clinical trials. Blinding was not a requisite for cryo-EM structural studies as the assay results are not subject to bias. During data processing, particles were grouped into two separate datasets to asses the resolution limit and preformed automatically by the program and does not imply any human supervision or selection during final refinement.

Reporting for specific materials, systems and methods

We require information from authors about some types of materials, experimental systems and methods used in many studies. Here, indicate whether each material, system or method listed is relevant to your study. If you are not sure if a list item applies to your research, read the appropriate section before selecting a response.

Materials & experimental systems

n/a	Involved in the study	
☒	☐	Antibodies
☐	☒	Eukaryotic cell lines
☒	☐	Palaeontology and archaeology
☒	☐	Animals and other organisms
☒	☐	Clinical data
☒	☐	Dual use research of concern

Methods

n/a	Involved in the study	
☒	☐	ChIP-seq
☒	☐	Flow cytometry
☒	☐	MRI-based neuroimaging

Eukaryotic cell lines

Policy information about cell lines and Sex and Gender in Research

Cell line source(s)	Sf9 purchased from Expression Systems LLC
Authentication	Authentication is carried out by the manufacture, and the successful protein expression from these strains validates their authenticity.
Mycoplasma contamination	No mycoplasma contamination was observed.
Commonly misidentified lines (See ICLAC register)	No commonly misidentified cell lines were used in the study.
