# Supplementary figures and images for: Structural basis of the human NAIP/NLRC4 inflammasome assembly and pathogen sensing
Source: Nat Struct Mol Biol. 2024 Jan 4;31(1):82–91. doi: 10.1038/s41594-023-01143-z (PMC10803261; doi:10.1038/s41594-023-01143-z)

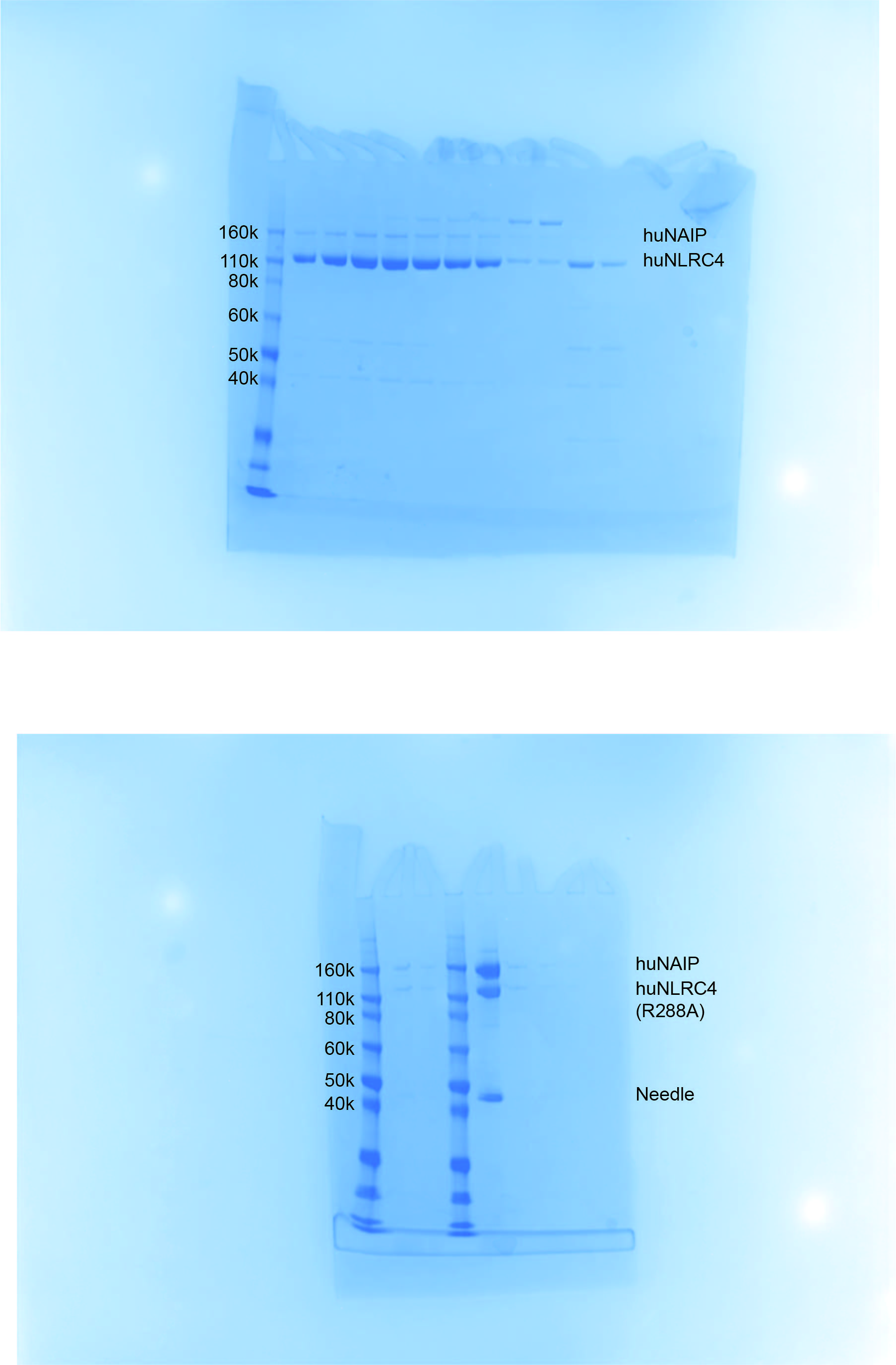

Supplement: Supplementary file 5 — Unprocessed SDS gels. [file 41594_2023_1143_MOESM5_ESM.jpg]
